# Comparative Analysis of Italian Lettuce (*Lactuca sativa* L. var. *ramose*) Transcriptome Profiles Reveals the Molecular Mechanism on Exogenous Melatonin Preventing Cadmium Toxicity

**DOI:** 10.3390/genes13060955

**Published:** 2022-05-26

**Authors:** Xuena Yu, Le Liang, Yongdong Xie, Yi Tang, Huaqiang Tan, Jianwei Zhang, Lijin Lin, Bo Sun, Zhi Huang, Ji Liu, Xiaomei Li, Lihua Tu, Huanxiu Li

**Affiliations:** 1 College of Horticulture, Sichuan Agricultural University, Chengdu 611130, China; yxn4842@sina.cn (X.Y.); 15294410767@163.com (L.L.); zhangjw1831@163.com (J.Z.); bsun@sicau.edu.cn (B.S.); dr.huangzhi@gmail.com (Z.H.); lxmwsl@126.com (X.L.); 2 College of Horticulture, Northwest A & F University, Xianyang 712100, China; 3 Chengdu Academy of Agriculture and Forestry Sciences, Chengdu 611130, China; xydcom@sina.cn (Y.X.); huaqiangtan@gmail.com (H.T.); liuji34905418@hotmail.com (J.L.); 4 Institute of Pomology and Olericulture, Sichuan Agricultural University, Chengdu 611130, China; tangyi@sicau.edu.cn (Y.T.); llj800924@sicau.edu.cn (L.L.); 5 Vegetable Germplasm Innovation and Variety Improvement Key Laboratory of Sichuan, Chengdu 610300, China; 6 College of Forestry, Sichuan Agricultural University, Chengdu 611130, China; tulhsicau@163.com

**Keywords:** cadmium resistance, comparative transcriptome, melatonin, lettuce breeding

## Abstract

Cadmium (Cd) accumulation in lettuce causes a large amount of yield loss during industry. Although many studies report that exogenous melatonin helps to alleviate the Cd stress of lettuce, the molecular mechanism for how plant tissue responds to Cd treatment is unclear. Herein, we applied both PacBio and Illumina techniques for Italian lettuce under different designed treatments of Cd and melatonin, aiming to reveal the potential molecular pathway of the response to Cd stress as well as the how the pre-application of exogenous melatonin affect this process. This result reveals that the root has the biggest expression pattern shift and is a more essential tissue to respond to both Cd and melatonin treatments than leaves. We reveal the molecular background of the Cd stress response in prospects of antioxidant and hormone signal transduction pathways, and we found that their functions are diverged and specifically expressed in tissues. We also found that candidate genes related to melatonin detoxify during Cd stress. Our study sheds new light for future research on how melatonin improves the cadmium resistance of lettuce and also provide valuable data for lettuce breeding.

## 1. Introduction

Cadmium (Cd) is a heavy metal that threatens human health globally; its accumulation in the human body causes disorders and increases the risk of cancer, Itai-Itai disease, and nephrotoxicity [1,2]. As with other heavy metals, Cd accumulates in the environment via mining, irrigation, sewage sludge application, rock phosphate fertilizers, and vehicular and industrial activities [3,4]. Cd is absorbed by vegetables from soil that is exposed to long-term contamination from nearby industry activities or mines, finally making its way into humans through the food chain [5,6]. Beyond its negative health implications, the accumulation of Cd toxifies the essential factors involved in the economic value of vegetables, such as the biomass, yield, and quality [7]. In recent years, the negative effects of Cd on many horticultural plants such as pepper [8,9], potato [10], tomato [11], pakchoi [12], and lettuce [13] have been revealed. These studies show various toxic phenotypes after Cd accumulation, such as the reduction in the length, surface area, and fresh weights of plant tissues such as leaves and roots. Together, the photosynthesis is also negatively affected, and it has been reported that the photosynthetic rate and contents of photosynthetic pigments decrease after a treatment of high Cd concentration [14]. Additionally, Cd stress blocks the normal uptake of developmentally crucial nutrient such as K (potassium), P (phosphorus), Ca (calcium) and Mn (manganese) [2,11]; this disruption can be attributed to the competition between Cd and other mineral nutrients around the root surface [5,6,7].

To promote the resistance of plant to cadmium stress, treatment of melatonin has been reported as an efficiency method [15]. Melatonin has emerged as a research highlight regarding its important role in regulating plant growth and adaptation to environmental stresses [16,17]. When suffering environmental stress, the level of melatonin increases in plants [18], and it was believed to act as a bio-stimulant or in enhancing the tolerance against abiotic stresses such as drought [19], extreme temperature [20,21], chemical stresses [22], and heavy metals [23,24]. The effect of exogenous melatonin on plants can also delay leaf senescence, improve the resistance to abiotic stress, and optimize growth parameters such as the seedling growth, leaf size, plant height, biomass, and pod and seed number [25]. It was reported that melatonin regulates the hormonal homoeostasis by interacting with other phytohormones such as cytokinin (CK), ABA, and GA to protect the plant from stress [26,27]. Actually, a lot of damage from abiotic stress is involved in the increase in the ROS level, and both endogenous and exogenous melatonin interacts with the stress signaling pathway by participating in the scavenging of ROS [28]. In most cases, melatonin elevates the transcript level of many antioxidative-related genes, such those encoding superoxide dismutase (SOD), ascorbate peroxidase (APX), catalase (CAT), and peroxidase (POD), facilitates the regeneration of endogenous antioxidants such as glutathione and vitamins C and E [28,29], and activates the antioxidant system AsA–GSH (reduced glutathione) cycle [30] and calcium-dependent signaling pathway [31]. In previous studies, it was reported that exogenous melatonin improves plant adaptation to Cd stress by decreasing Cd accumulation and re-establishing ROS homeostasis [32]. In tomato, the exogenous melatonin alleviated the Cd stress by improving the detoxication activity [23], and the chlorophyll content and photosynthetic efficiency are also reduced by the decrease in the melatonin level under Cd stress [33]. In wheat, melatonin treatment triggers a significant increase in glutathione peroxidase (GSH) content, and the activities of APX and SOD were also specifically improved. In addition, the genes involved in tolerance to Cd toxicity were upregulated after melatonin treatment in Arabidopsis, such as ABC transporter and PCR2 in alfalfa seedlings, and PDR8 and HMA4 [32,34,35].

Lettuce is one of the most popular leafy vegetables and occupies a large proportion of the worldwide agricultural industry [36]. It has a significantly high capacity for Cd accumulation in its tissues and is characterized as an important model to study the mechanisms that are responsible for the accumulation of Cd [37]. Under Cd stress, the fresh and dry matter yield of leaves and roots was significantly reduced in lettuce, and both roots and leaves show an increase in H_2_O_2_ content while the superoxide dismutase SOD was stimulated [13,38,39]. After melatonin treatment, the photosynthetic rate, stomatal conductance, and light-use efficiency were more improved than those of the control [40]. Additionally, exogenous melatonin could eliminate the excess nitrate stress in lettuce tissues and enhance the photosynthesis [41]. Although Cd accumulation is a serious threat to the lettuce industry, work focusing on how melatonin alleviates Cd stress in lettuce is still insufficient. Only a few studies have been conducted to explore how melatonin treatment influences the abiotic-stress resistance of lettuce. Thus, a comprehensive study on how melatonin alleviates Cd stress in lettuce is needed, and the corresponding molecular mechanism needs to be illustrated.

**Figure 1:**
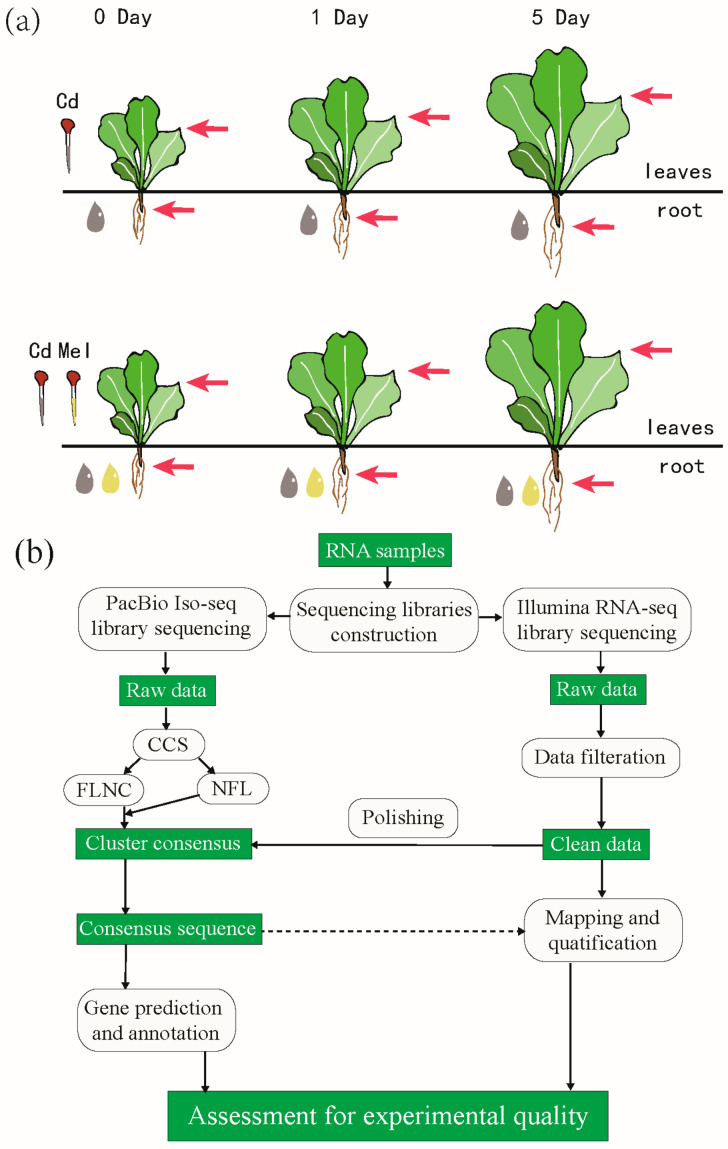
Experimental processing and RNA-seq. (**a**) Sample treatment. The upper picture shows lettuce under cadmium stress, and the lower picture shows cadmium stress of lettuce pre-treated with melatonin. (**b**) Library construction and sequencing.

Transcriptome comparison between seedling with or without melatonin pretreatment under Cd stress is an appropriate way to detect the relevant response molecular pathway and improve our understanding on Cd resistance in lettuce. To illustrate how these processes are influenced by melatonin, we designed several comparative groups under different Cd and melatonin treatments on both leaves and roots and obtained their transcriptomes over time by next-generation sequencing (Figure 1, Table S1). We also applied single-molecule real-time sequencing to obtain a high-quality reference transcriptome and calculated the expression profiting. After analyzing several important pathways related to Cd resistance, we attempted to answer the following issues: (1) how the molecular networks respond to Cd stress in lettuce; (2) how applied melatonin eliminates Cd toxicity in lettuce; (3) what the difference for transcriptome profiling of leave and root is when treated by exogenous melatonin. In this study, we provided the first transcriptome study for Cd and melatonin treatment in lettuce, which provides the foundation for further exploration of the molecular mechanism of Cd accumulation, and develop breeding strategies aimed at decreasing Cd in crop plants. This work will improve our understanding of the protective effects of exogenous melatonin on plants under Cd stress.

## 2. Materials and Methods

### 2.1. Sample Preparation and Treatment

Italian lettuce (*Lactuca sativa* L. var. *ramosa* Hort.) is one of the most popular leafy vegetables in China. Italian Lettuce seeds were sterilized with 10% hydrogen peroxide for 10 min, rinsed thoroughly with distilled water three times, then germinated on moist filter paper in the dark at 20 °C. After the initiation for 0.5 cm, the seeds were transferred to a salver (40 × 30 cm) filled with perlite and covered by vermiculite for moisture retention. Then, they were transferred into an incubator under 23 °C/18 °C in a 14 h/10 h day/night cycle, respectively. The relative humidity was set at 70%, and the light intensity was 15,000 l ×. After the formation of seedlings, they were watered with 200 mL half-strength Hoagland’s nutrient solution each day for 20 days, and subsequently cultivated in a growth chamber. Twenty days later, the seedlings were divided into three groups: (1) seedlings without any treatment refer to the control group; (2) seedlings cultivated with the nutrient solution with 1 μmol/L melatonin (Sigma-Aldrich, St. Louis, MO, USA); (3) seedlings cultivated with the nutrient solution without melatonin two days later. Then, the latter two groups were treated with 50 μmol/L CdCl_2_ for simulation of a Cd stress environment, and the nutrient solution was replenished once for three days to maintain constant concentrations. There were 20 seedlings per treatment, repeated 3 times. The physiological parameters of three groups for both their leaves and roots were estimated 0, 1, 3, 5, and 10 days after Cd treatment. The growth-factor estimation was only conducted on group 2 and group 3. The leaves and roots of 0-, 1-, and 5-day-old seedlings of group 2 and group 3 were sampled for transcriptome sequencing, and three biological repeats were included for each treatment (Table S1). Except for their use in conductivity measurement, all samples were stored in by ultra-low-temperature freezing under −80° before conducting.

### 2.2. Library Construction and Sequencing

Total RNAs for samples were extracted by RNA extraction tool kit “RNAiso-plus” following the manufacturer’s recommendation. RNA degradation and contamination was monitored on 1% agarose gel and was then checked using a NanoPhotometer^®^ spectrophotometer (IMPLEN, Westlake Village, CA, USA). A total amount of 3 μg RNA per sample was used as input material for the RNA sample preparations. Sequencing libraries were generated using NEBNext^®^ UltraTM RNA Library Prep Kit for Illumina^®^ (NEB, Ipswich, MA, USA) following the manufacturer’s recommendations, and index codes were added to attribute sequences to each sample. Briefly, mRNA was purified from total RNA using poly-T oligo-attached magnetic beads. Fragmentation was carried out using divalent cations under an elevated temperature in NEBNext First Strand Synthesis Reaction Buffer (5X). First-strand cDNA was synthesized using random hexamer primer and M-MuLV Reverse Transcriptase (RNase H-). Second-strand cDNA synthesis was subsequently performed using DNA Polymerase I and RNase H. The remaining overhangs were converted into blunt ends via exonuclease/polymerase activities. After adenylation of 3′ ends of DNA fragments, an NEBNext Adaptor with a hairpin loop structure was ligated to prepare for hybridization. In order to select cDNA fragments of, preferentially, 250~2300 bp in length, the library fragments were purified with an AMPure XP system (Beckman Coulter, Beverly, USA). Then, 3 μL USER Enzyme (NEB, USA) was used with size-selected, adaptor-ligated cDNA at 37 °C for 15 min followed by 5 min at 95 °C before PCR. Then, PCR was performed with Phusion High-Fidelity DNA polymerase, Universal PCR primers, and Index (X) Primer. Finally, PCR products were purified (AMPure XP system) and the library quality was assessed on an Agilent Bioanalyzer 2100 system. Clustering of the index-coded samples was performed on a cBot cluster-generation system using TruSeq PE Cluster Kit v3-cBot-HS (Illumia) according to the manufacturer’s instructions. After cluster generation, the library preparations were sequenced on an Illumina Hiseq platform, and 125 bp/150 bp paired-end reads were generated.

The Iso-Seq library was prepared according to the Isoform Sequencing protocol (Iso-Seq) using the Clontech SMARTer PCR cDNA Synthesis Kit and the BluePippin Size Selection System protocol as described by Pacific Biosciences (PN 100-092-800-03).

Raw data (raw reads) of fastq format were first processed through in-house perl scripts. In this step, clean data (clean reads) were obtained by removing reads containing the adapter, reads containing ploy-N, and low-quality reads from raw data. At the same time, from the Q20, Q30, and GC content, clean data were calculated. All the downstream analyses were based on the clean data with high quality.

### 2.3. Subread Processing and Error Correction

Sequence data from Iso-Seq library were processed using the SMRTlink v5.0 software [42]. Circular consensus sequence (CCS) was generated from subread BAM files, parameters: min_length 200, max_drop_fraction 0.8, no_polish TRUE, min_zscore −999, min_passes 1, min_predicted_accuracy 0.8, max_length 18000. CCS.BAM files were output, which were then classified into full length and non-full length reads using pbclassify.py, ignorepolyA false, and minSeqLength 200. Effective subreads were obtained by the SMRT Link v5.0 under the parameter: MinLength = 200, MinReadScore = 0.75. Then, the CCS was obtained from Iso-Seq pipeline using the parameter MinPasses = 1 and MinPredictedAccuracy = 0.8 After examining for poly(A) signal and 5′ and 3′ adaptors, only the CCS with all three signals was considered as an FLNC read. The non-full-length and full-length fasta files produced were then fed into the cluster step, which performs isoform-level clustering (ICE), followed by final Arrow polishing, hq_quiver_min_accuracy 0.99, bin_by_primer false, bin_size_kb 1, qv_trim_5p 100, and qv_trim_3p 30. Finally, LoRDEC was used to correct the consensus transcripts based on all transcriptomes from the illumine short-read sequencing dataset. Then, CD-HIT-v4.6 [34] was used to remove the redundancy of FLNC.

### 2.4. Transcriptome Characterizing and Annotation

The error-corrected FLNC reads were mapped to the genome sequence of the lettuce genome (*L*. *sativa* Lsat Salinas v7) using GMAP as described previously. We used the no-chimera setting to ensure mapping on the same contig as much as possible. The best mapped locus was chosen for each FLNC read based on both the identity and coverage values. The mapping results were divided into three categories: known genes, novel isoform, and novel genes. The genome mapping results of FLNC reads were visualized using the Integrative Genome Viewer.

SUPPA was applied to obtain the alternative splicing (AS) events. The analysis of fusion genes and lncRNAs was performed with CPC, CNCI, CPAT, and Pfam software, according to the respective instructions. As lncRNAs do not encode proteins, the transcript can be screened to determine whether it has the potential of coding ability. If one has no potential coding ability, it would be categorized as a bona-fide lncRNA. Four coding potential analysis methods were used to predict the lncRNAs. These methods included Coding Potential Calculator (CPC) [43], Coding-Non-Coding Index (CNCI) [44], Pfam (https://pfam.xfam.org/ accessed on 4 May 2020), and Predictor of long non-coding RNAs and messenger RNAs based on an improved k-mer scheme (PLEK) [45]. TAPIS pipeline software was used to identify alternative polyadenylation (APA). The features illustrated by Circos. The ANGEL pipeline was used in order to determine protein coding sequences from cDNAs. We used this species or closely related species for confident protein sequences for ANGEL training and then ran the ANGEL prediction for the given sequences. Transcription factors (TF) were predicted using iTAK software. The SSR of the transcriptome were identified using MISA, which allows the identification and localization of perfect microsatellites as well as compound microsatellites, which are interrupted by a certain number of bases.

Gene function was annotated by BLAST software based on the following databases: Nr (NCBI non-redundant protein sequences), Nt (NCBI non-redundant nucleotide sequences), Pfam (Protein family), KOG/COG (Clusters of Orthologous Groups of proteins), Swiss-Prot (A manually annotated and reviewed protein sequence database), KO (KEGG Ortholog database), and GO (Gene Ontology).

### 2.5. Differential Expression Analysis and Function Enrichment

The consensus Iso-RNA transcripts were used as a reference for expression estimation. An index of the reference was built using Bowtie v2.2.3, and paired-end clean reads of HiSeq data were aligned to the reference using TopHat v2.0.12. To estimate the expression level, HTSeq v0.6.1 was used to count the read numbers mapped to each gene. Then, the FPKM of each gene was calculated based on the length of the gene and reads count mapped to this gene. FPKM refers to the expected number of fragments per kilobase of transcript sequence per millions base pairs sequenced, which considers the effect of the sequencing depth and gene length for the read count at the same time and is currently the most commonly used method for estimating gene expression levels [46].

Differential expression analysis between each two conditions (two biological replicates per condition) was performed using the DESeq R package (1.18.0). DESeq provides statistical routines for determining differential expression in digital gene expression data using a model based on the negative binomial distribution. The resulting P-values were adjusted using the Benjamini–Hochberg approach for controlling the false-discovery rate. Genes with an adjusted *p*-value < 0.05 found by DESeq were assigned as differentially expressed.

Gene-ontology (GO) enrichment analysis of differentially expressed genes was implemented by the GOseq R package, in which gene length bias was corrected. GO terms with a corrected *p*-value less than 0.05 were considered significantly enriched by differentially expressed genes. KEGG is a database resource for understanding high-level functions and utilities of the biological system, such as the cell, the organism, and the ecosystem, from molecular-level information, especially large-scale molecular datasets generated by genome sequencing and other high-throughput experimental technologies (http://www.genome.jp/kegg/, accessed on 10 May 2020). We used KOBAS software to test the statistical enrichment of differential expression genes in KEGG pathways.

## 3. Results

### 3.1. Transcriptome Features and Description

**Figure 2:**
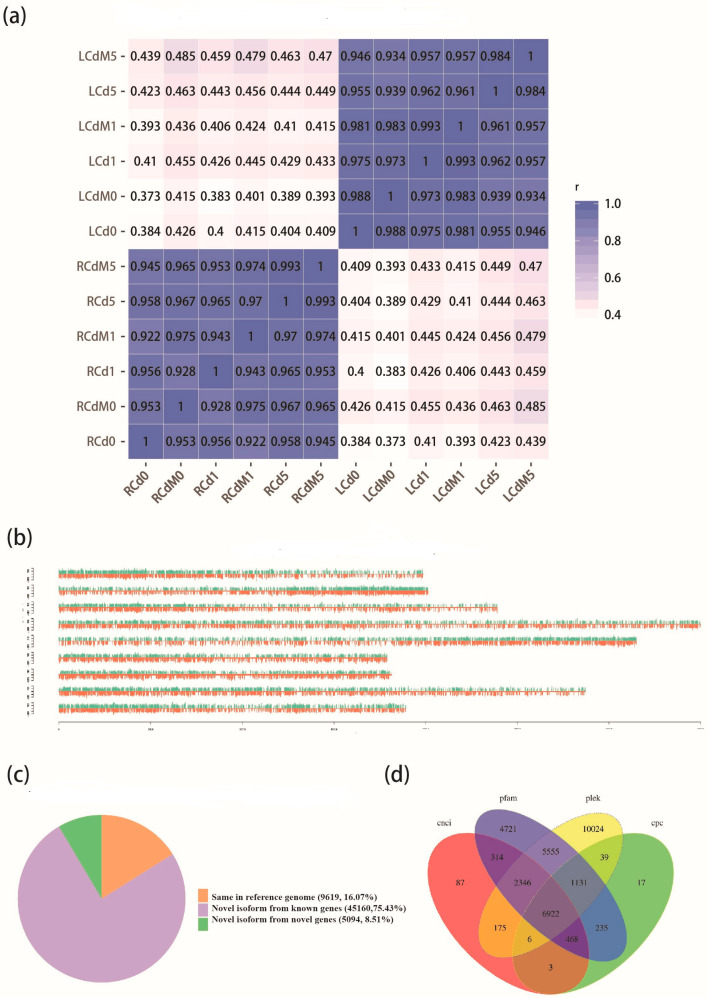
Assess experimental data quality. (**a**) Pearson correlation between samples (*p*-value < 0.05); (**b**) read density in chromosomes; (**c**) classification transcripts correlated by genome; (**d**) Venn diagrams of lncRNAs predicted by four coding potential analysis methods. These methods included Coding Potential Calculator (cpc), Coding-Non-Coding Index (cnci), Pfam and Predictor of long non-coding RNAs and messenger RNAs based on an improved k-mer scheme (plek).

To explore the dynamic gene expression pattern for the roots and aboveground part of lettuce under various treatments, 36 transcriptomes representing 12 treatment groups (three biological repeats for each) were sequenced by Illumina RNA-seq (Figure 1). We obtained 269.55 Gb of clean data (GC content between 43.65% and 45.24%, Table S2), and the total amount of paired-end reads ranged from 43,514,916 to 70,524,938 per sample. The Pearson’s correlation coefficients in correlation analysis are higher than 0.94 on all samples, indicating that the high repetition rate for each group and could be used in downstream analysis (Figure S1), and the values are similar among leaves or roots separately (Figure 2a). The data were upload to the NCBI database under SRA accession PRJNA597399.

Due to the lack of reference genome for Italian lettuce (*L*. *sativa* L. var. *ramose* Hort.), a high-quality reference transcriptome is required for further expression analysis. We applied PacBio Iso-Seq platform to obtain the long-read transcriptome for Italian lettuce. We obtained 14.12 Gb of PacBio reads, finally resulting in 9,228,516 subreads after filtering. The average length of these subreads was 1531 with N50 = 3512. In total, we obtained 595,853 circular consensus sequences (CCSs), including 438,052 full-length non-chimera (FLNC) with an average length of 3007 since each of them contained a distinct poly (A) tail and 5′ and 3′ cDNA synthesis primers. Finally, we obtained 213,865 consensus reads after ICE modification. To further correct these consensus reads, the Illumina HiSeq 2000 transcriptomic reads for all treatments have been applied to perform the polishing, and finally obtained the same number of consensuses reads as 213,865 while the contig N50 increased from 4782 to 4839 bp (Table S2). Among 213,865 consensus reads, 88.38% (189,010) can be mapped onto the lettuce reference genome (*L*. *sativa* L.) [47]. A total of 187,452 reads were multiple mapped result, while the uniquely mapped occupied 0.73% of the total data. The reads that could only be mapped onto “+” or “−” chains were both less than 1% (Figure 2b). This result indicated the completion and correction of the consensus reads. Then, CD-HIT v. 4.6 [34] was used to remove the redundancy of consensus reads, resulting in 59,873 transcripts as the final reference transcriptome.

Compared to the annotation of the reference genome, we identified 45,160 novel transcripts for the known genes in the reference genome and 3153 novel genes (Figure 2c, Tables S3 and S4). For a total of 3153 novel genes, 62.99% (1986) were annotated in at least one of the following six databases: Cluster of Orthologous Groups (COG) of proteins, the gene ontology (GO), the Kyoto Encyclopedia of Genes and Genomes (KEGG), the protein family (Pfam), the manually annotated and reviewed protein sequence database (SwissProt), and non-redundant protein (NR) databases (Table S3). We found a total of 5917 alternative splicing (AS) events (Table S5). Since alternative splicing events can be divided into five main categories, alternative 3′ splice sites (A3SS), alternative 5′ splice sites (A5SS), retained introns (RI), skipped exons (SE), mutually exclusive exons (MXE), alternative first exon (AF), and alternative last exon (AL), we found that the most frequent AS event is RI (2490), followed by A3 (1591) and A5 (1000). The MX was the least frequent AS event, and we only detected 19 in total. Furthermore, a total of 3209 genes were annotated as encoding transcription factors (TFs) representing 89 families. In these TF families, SNF2 occupied the largest number while PHD and SET were the second- and third-largest groups, respectively.

A fusion gene is a chimeric gene composed of two or more separate genes. We identified a total of 258 fusion genes in our SMRT-seq result, and the details can be found in Table S6. A total of 10,948 alternative polyadenylation (APA) genes were identified. Among them, 6000 genes (54.8%) with a single poly-A site were detected, and 2500 genes had at least two poly-A sites. For lncRNAs, we identified 8821 in CPC, 10,322 in CNCI, 26,198 in PLEK, and 21,691 in Pfam. A total of 6922 lncRNAs were annotated among all datasets (Figure 2d, Tables S7–S10). Within them, 6575 lincRNAs, 199 antisense-lncRNAs, and 88 intronic-lncRNAs were identified.

### 3.2. Expression Pattern during Cd Stress and Melatonin Treatment

**Figure 3:**
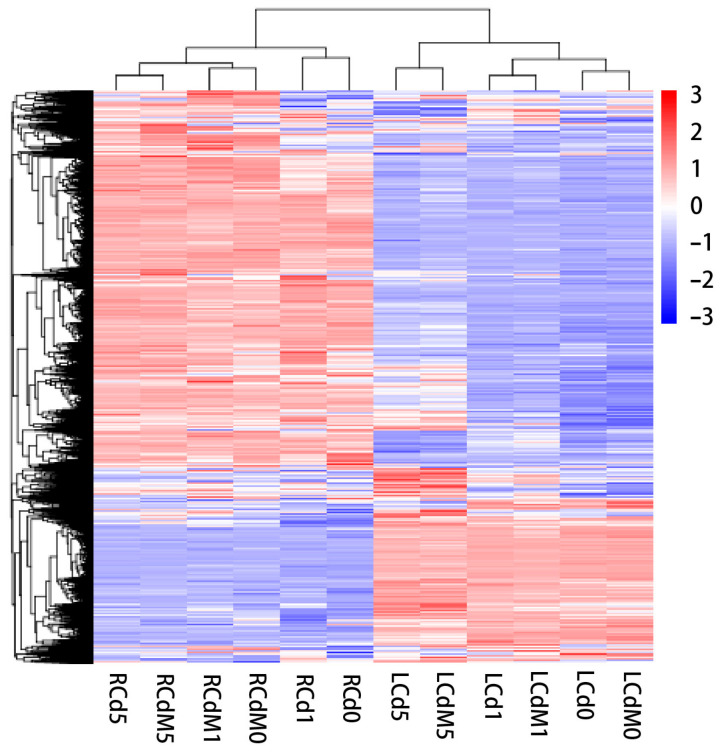
Comparison group differential gene expression heatmap. Leaf samples on days 0, 1, and 5 under cadmium stress are represented by LCd0, LCd1, and LCd5. Leaf samples on days 0, 1 and 5 under cadmium stress that were pre-treated by melatonin are represented by LCdM0, LCdM1, and LCdM5. Root samples on days 0, 1, and 5 under cadmium stress are represented by RCd0, RCd1, and RCd5. Root samples on days 0, 1, and 5 under cadmium stress that pre-treated by melatonin are represented by RCdM0, RCdM1, and RCdM5.

To study the molecular mechanism involved in Cd or melatonin responses in lettuce, we defined six comparing groups to detect the DEGs (Figure 3 and Table 1): Group 1 (G1) contains the root samples under Cd stress in the beginning (day 0), first day (day 1) and fifth day (day 5), which was used to illustrate the molecular process invoked by lettuce to respond to Cd in the roots over time. Group 2 (G2) contained the samples under Cd stress pre-treated by melatonin at 0, 1, and 5 days; this group was used to study how the expression patterns change with melatonin treatment under Cd stress over time. Group 3 (G3) was used to compare the expression patterns between samples with or without melatonin treatment at each time point (day 0, day 1, or day 5), which was used to study how melatonin treatment affects the pathway related to Cd resistance. Groups 4–6 (G4–6) one-to-one corresponded to the comparisons of Groups 1–3 in the aboveground part of lettuce. In G1, there was a larger amount of DEGs between day 0 and day 1 than between day 1 and day 5 (1746 and 474). From day 0 to day 1, 752 genes were upregulated and 994 were downregulated. From day 1 to day 5, 282 genes were upregulated and 192 were downregulated, respectively. In G2, 177 genes were upregulated and 326 were downregulated between day 0 to day 1, and 246 and 84 were in comparison between day 1 and day 5. This result shows that day 1 demonstrated a stronger shift under Cd stress. G3 comparison demonstrates the large differences of expression patterns after melatonin treatment in the roots. At day 0, 1140 DEGs were identified between samples with or without melatonin treatment. On the first day, the number increased to 3527, which seems like a “response peak” after applied melatonin. No DEGs were identified on the fifth-day comparison. G4 to G6 reflect the expression pattern in the aboveground part. In G4, there were 777 DEGs between day 0 and day 1, and 1484 between day 1 and day 5, respectively. In G5, the number of DEGs was much less than the G4 group without melatonin pretreatment, in which 91 genes were upregulated and 80 were downregulated between day 0 to day 1, and 405 were upregulated and 536 genes were downregulated between day 1 and day 5. Both trends indicate that the above part in lettuce has a stronger response to Cd stress later than in the roots. G6 comparison demonstrates less difference in the expression pattern; we only detected 30 DEGs on the day 0 comparison, and 17 DEGs on the day 5 comparison. There was no DEG on the day 1 comparison.

In the melatonin treatment groups (G2 and G5), the DEGs are significantly lower than in the groups without melatonin treatment (G1 and G4) over time in both the roots and leaves. Thus, the pattern suggests the possibility that the melatonin eased at least part of the Cd damage by alleviating the oxidative stress. Then, we focused on the G3 and G6 comparisons, which more explicitly show the differences between lettuce with or without melatonin treatment. One day after Cd stress, the roots of lettuce pretreated by melatonin showed a significantly different gene expression pattern compared to the control, which demonstrates more than 3000 DEGs, while this difference disappeared on the fifth day (Table 1). This result also supported that the roots with melatonin treatment in lettuce have strong metabolic activity compared to the control. In addition, comparing the sample between with or without melatonin treatment at a given time point, the influence induced by applied melatonin was mostly apparent in the beginning and was demonstrated in the roots, while the leaves showed a weak melatonin response across all timepoints. This evidence indicates that the applied melatonin is most functioning in the roots at first and the influence suddenly decreases from day 1 to day 5 for lettuce, while the applied melatonin in the leaves has a weak effect on Cd erasure.

### 3.3. Functional Enrichment during Cd Response and Melatonin Treatment

To further illustrate the genes related to the Cd response as well as the melatonin, we conducted both GO and KEGG enrichments on the DEGs. We first focused on the genes activated during Cd stress (G1 and G4). In the initial stage of the Cd accumulation in the roots (day 0 to day 1), the upregulated DEGs were involved in transmembrane transporter and metal ion transport, including genes encoding ABCC2, PHO84, zinc transporters (SLC39A1_2_3), and copper transporters (CTR1). The downregulated genes are relevant to cellulose metabolism, hydrolase activity, and energy metabolism, including genes encoding cellulose synthases (CESA) and xyloglucosyl transferase. Terms related to the oxidation-reduction process were detected in both up- and downregulated genes (Table S12). In upregulated DEGs of oxidation reduction, we found genes encoding lipoxygenase (LOX), peroxidase (POX), thioredoxin 1 (trxA), and SOD2 (superoxide dismutase, Fe-Mn family). In downregulated DEGs, we also found genes encoding SOD2 and POX, indicating the division of labor. Between day 1 and day 5, the upregulated genes were mostly involved in the oxidation-reduction process, hydrolysis, copper ion binding, and energy metabolism, which also included genes encoding POX, laccase, and pectinesterase. Moreover, the genes related to superoxide metabolism, metal ion binding, ammonium transport, and response to abiotic stress were downregulated, in which we found genes encoding SOD1 (superoxide dismutase, Cu-Zn family), CCS (copper chaperone for superoxide dismutase), and also LOX. In the roots, since the Cd accumulation directly induced the oxidative-stress status, the genes related to oxidation-reduction processes were upregulated in response to the Cd stress, and the genes involved in hydrolase activity and cellulose metabolic also changed due to the high content of ROS. From the first to fifth day, the genes involved in superoxide metabolism and response to abiotic stress were upregulated, indicating the continuous damage of Cd accumulation. Although the Cd stress was precepted in the roots, the upregulated DEGs between day 0 and 1 in the leaves were related to common biological processes. Until the fifth day, the upregulated GO terms in the leaves were relevant to detoxification, responding to stimulus and autophagosome, and were essential pathways in the abiotic stress resistance. In the aboveground part, the significantly enriched GO terms for upregulated genes were about the common biological processes such as transcription, translation, and energy metabolism from day 0 to 1, while the downregulated genes can be classified as the oxidation-reduction process, photosynthesis, and energy metabolism (Table S13). The downregulated genes included SOD2 and genes encoding the subunit of the photosystem such as *petA*, *psaE*, *psaL*, and *psbW*. From day 1 to day 5 after Cd stress, genes involved in ion binding, detoxification, response to stimuli, and autophagosome were upregulated, containing ATG8, calcium-dependent protein kinase (CPK), and calcium-binding protein (CML), and the genes with terms involved in transcription and translation were downregulated. The response was quite late in the leaves, and the time gap for the Cd transport from the roots to leaves was also reflected on the molecular scale.

To illustrate the gene-expression pattern in samples pre-treated by melatonin over time, we also checked the DEGs of comparison groups G2 and G5. On the first day after melatonin treatment in the roots, the enriched upregulated DEGs were related to the transmembrane, metal ion binding, and transport and photosynthesis. No gene encoding SOD was found. Between day 1 and day 5, the upregulated DEGs enriched into terms involved in ion transport (e.g., calcium-binding protein, CML, and SOD2) and protein processing, while those involved in the superoxide metabolic process (genes encoding SOD1, and CCS) and response to hormone (SAUR family protein) were downregulated (Table S14). In the roots, the comparison of day 0 to day 1 demonstrated that the upregulated DEGs were enriched in terms involved in plant hormone signal transduction, chitinase activity, oxidoreductase activity, and response to stress, including genes encoding chitinase and basic endochitinase B (CHIB) (Table S15). The downregulated genes were enriched in terms related to intramolecular lyase activity, antioxidant activity, and the oxidation-reduction process. These genes encode laccase, but not SOD, POX, or trxA. In the comparison between day 1 and day 5, the upregulated genes are involved in transcription and translation, kinase activity, plant hormone signal transduction, and oxidoreductase activity, while no SOD, POX, or trxA were detected as DEGs, while the enriched terms for downregulated genes were relevant to single-organism biosynthetic, photosynthesis, organonitrogen-compound-related process. This result suggests that melatonin treatment limited the Cd damage in the first stage, where no GO or KEGG terms related to enriched antioxidant pathways.

To assess whether the molecular pathways differed between samples with or without melatonin pretreatment on day 0, day 1, and day 5, we checked the enrichment for G3 and G6 comparative groups (Tables S16 and S17). In the roots, the number of DEGs was quite large between samples with or without melatonin treatment, indicating a stronger influence by applying melatonin. From day 0, the number of DEGs reached the highest on day 1 (3527 in total) and decreased to 0 on day 5. In the day 0 comparison, many pathways related to the stress response were downregulated with the melatonin treatment, such as the terms “response to oxidative stress” and “response to stress”. Until day 5, it related to “TOR signaling”, “autophagosome assembly”, and “macroautophagy”. The upregulated DEGs were related to the cell-wall macromolecular process, chitin catabolic (chitinase, basic endochitinase B, CHIB) or hormone-related process (ERS ethylene receptor, ETR), photosystems (*psaN*, *psaE*, *psaH*, *psaL*, *psaD*, *psbY*, *psbP*, *psbW* and *psbR*), ion binding and transporter, and oxidoreductase activity (laccase, trxA, POX). At the same time, the downregulated GO terms were related to POX activity, the oxidation-reduction process, integral component of mitochondrial membrane, response to stress, and nicotianamine synthase activity, which included genes encoding AMT, MEP ammonium transporter, POXs, heat-shock 70 kDa protein 1/8 (HSPA1_8), and laccase. In GO enrichment of day 1, the upregulated DEGs are enriched into the terms involved in phosphorylation, hydrolase activity, transferase activity, cellulose and cell wall, ion binding, steroid dehydrogenase activity, and energy metabolism; and the corresponding KEGG terms are related to plant hormone signal transduction, biosynthesis of secondary metabolites, and energy metabolism. These upregulated DEGs consisted of genes encoding cellulose synthase A (CESA), pectinesterase, interleukin-1 receptor-associated kinase 4 (IRAK4), laccase and chitinase. For the genes downregulated after melatonin treatment for one day, the enriched GO terms are related to ion binding and transport, oxidoreductase activity, the trehalose metabolic process, ion channel activity, autophagy, and TOR signaling. The KEGG terms are hormone signal transduction, linoleic acid metabolism, photosynthetic organisms, carbon metabolism, and plant–pathogen interaction. On day 5, no DEGs were identified. In the leaves, on day 0, the upregulated GO terms related to energy metabolism and transcriptional activity, while the genes with terms involved in ion transport (solute carrier family 39 (zinc transporter)) were downregulated. In KEGG enrichment, only “Galactose metabolism” was significantly enriched for upregulated genes and only “Plant hormone signal transduction” for downregulated genes. No DEGs were recognized in the day 1 comparison in the leaves. On day 5, the enriched GO terms for upregulated genes were involved in the response to stimulus and cytokine receptor binding and are relevant to energy metabolism and cellulose synthesis for downregulated genes. In KEGG enrichment, only “Circadian rhythm” was enriched for upregulated genes, and no term was significantly enriched for the downregulated gene set. In the roots pre-treated by melatonin, the genes involved in the molecular pathway related to the cell-wall macromolecule metabolic process, photosynthesis, and energy metabolism have significantly higher levels than samples without melatonin, and the comparison on day 1 also shows the higher expression level of genes involved in energy metabolism and housekeeping biological processes such as phosphorylation and protein kinase activity (Table 1).

### 3.4. Functional Divergence of Genes Involved in Antioxidant System

**Figure 4:**
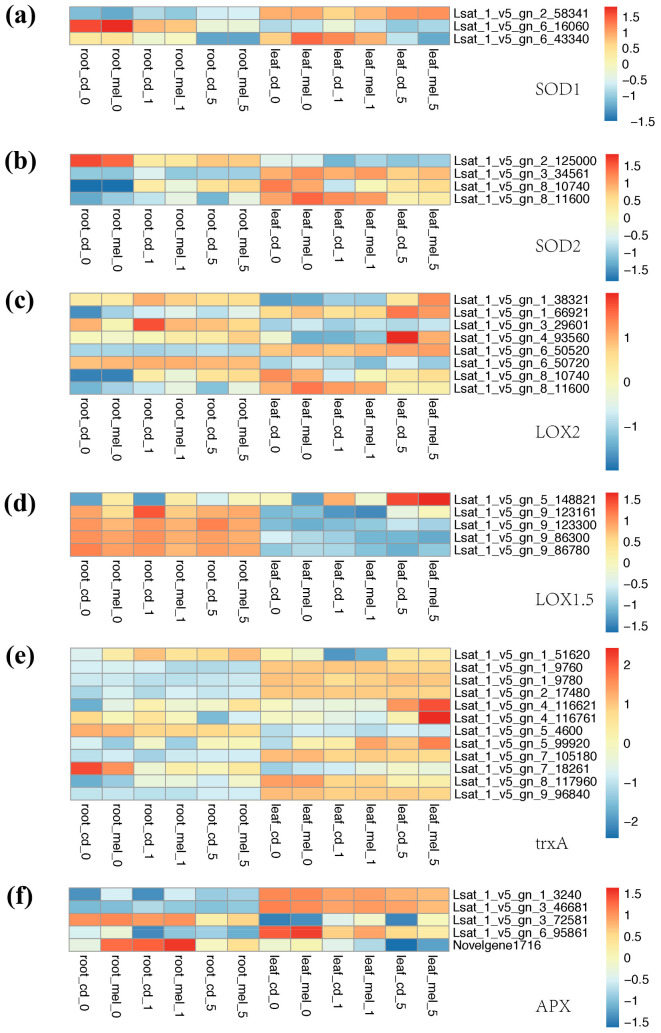
Antioxidant pathway-related gene expression heat map. Leaf samples on days 0, 1, and 5 under cadmium stress are represented by leaf_cd_0, leaf_cd_1, and leaf_cd_5. Leaf samples on days 0, 1, and 5 under cadmium stress that were pre-treated by melatonin are represented by leaf_ mel_0, leaf_ mel_1, and leaf_ mel_5. Root samples on days 0, 1, and 5 under cadmium stress are represented by root_cd_0, root_cd_1, and root_cd_5. Root samples on days 0, 1, and 5 under cadmium stress that were pre-treated by melatonin are represented by root_mel_0, root_mel_1, and root_mel_5. (**a**) *SOD1*; (**b**) *SOD2*; (**c**) *LOX2*; (**d**) *LOX1.5*; (**e**) *trxA*; (**f**) *APX*.

**Figure 5:**
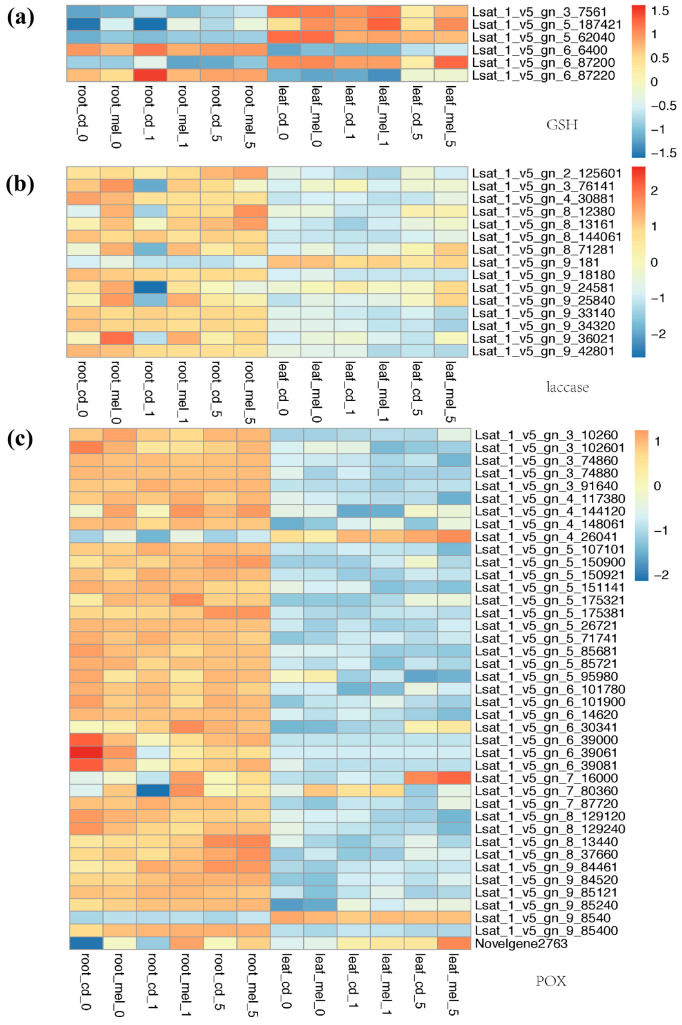
*GSH*, *laccase* and *POX* gene expression heat map. Leaf samples on days 0, 1, and 5 under cadmium stress are represented by leaf_cd_0, leaf_cd_1, and leaf_cd_5. Leaf samples on days 0, 1, and 5 under cadmium stress that were pre-treated by melatonin are represented by leaf_ mel_0, leaf_ mel_1, and leaf_ mel_5. Root samples on days 0, 1, and 5 under cadmium stress are represented by root_cd_0, root_cd_1, and root_cd_5. Root samples on days 0, 1, and 5 under cadmium stress that were pre-treated by melatonin are represented by root_mel_0, root_mel_1, and root_mel_5. (**a**) *GSH*; (**b**) *laccase*; (**c**) *POX*.

To reveal how lettuce responds to the ROS accumulation due to the Cd treatment and the molecular background that the melatonin alleviates Cd stress, we looked at the genes related to the antioxidant pathways (Figure 4 and Figure 5), including homologs of SOD1, SOD2, LOX2, LOX1.5, trxA, and APX. Three copies of SOD1 show different expression patterns, 2_58341 had stronger expression in the leaves, while 6_16060 was strongly expressed in the roots. 6_43340 was strongly expressed in the first two days and later decreased. SOD2 also shows a different expression pattern between the roots and leaves. Both SOD2 and SOD1 have specific expressions in the roots or leaves; however, no significant change has been identified after melatonin treatment. LOXs play a key role in defense responses against biotic and abiotic environmental stresses; we also found that genes encoding LOX display a different expression pattern between samples. Most of these different expression LOX genes show a higher expression level in the roots; we found that a LOX1/5-like gene, 5_148821, was strongly induced by melatonin, and it showed a significantly higher expression level in melatonin treatment in both the roots and leaves. We found 12 trxA-like genes had different levels of expression between samples, several of them specifically highly expressed in the leaves, while 5_4600 had a higher expression level in the roots than leaves. In the roots, 7_18261 showed an extremely high abundance on day 0 after Cd treatment, indicating that this gene was the candidate with a function in early response to Cd stress in the roots. Five APX-like genes were identified as different expression genes, while also showing a divergent expression pattern between the roots and leaves. We found a gene annotated as a novel gene in Italian lettuce, *Novelgene1716*, that has a higher expression level in roots pre-treated by melatonin from day 0 to day 5. This pattern demonstrates that the expression level of APX-like gene was induced by melatonin treatment despite the expression level also having a rapid rise on day 1 after Cd treatment.

We found six GSH-like genes differentially expressed between samples; four copies have an expression bias in the leaves, while two have a higher FPKM value in the roots. Within these genes, 5_187421 has a higher expression level in each comparison in melatonin-treatment samples compared to the one only under Cd stress, which was a potential candidate for genes induced by melatonin. For genes encoding laccase, in the roots, four copies (2_125601, 8_12380, 8_71281, 9_36021) had steadily higher expression levels in the roots with melatonin treatment on day 0, 1 and 5 compared to relevant samples under Cd stress. The expression levels of 3_76141 and 9_24581 were upregulated by melatonin only on day 0 and day 1. There was a large number of POX-like genes showing different expression patterns between samples in lettuce. Within them, most of the copies had significantly higher FPKM values in the roots, except for 4_26041 and 9_8540. We found that *7_80360* and a gene annotated as *Novelgene2763* have upregulated RPKM values after melatonin treatment in both the roots and leaves.

### 3.5. Functional Divergence of Genes Involved in Hormone Signaling Transduction

**Figure 6:**
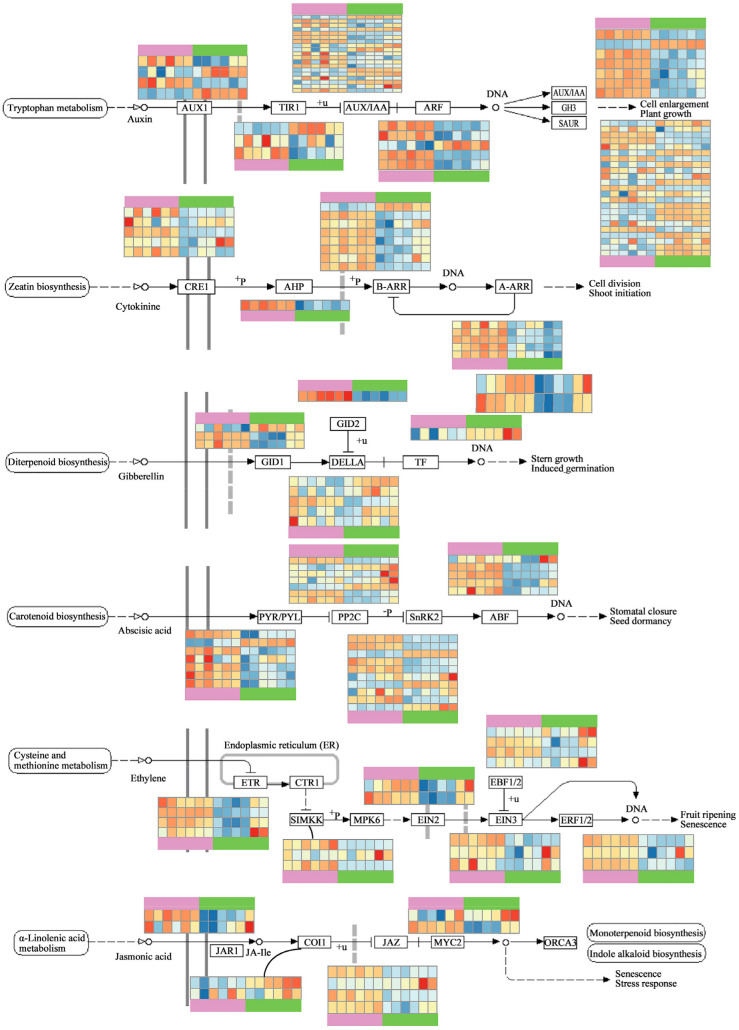
Expression patterns of genes in plant hormone signal transduction pathways. From top to bottom in the figure are tryptophan metabolism, zeatin metabolism, diterpenoid biosynthesis, carotenoid biosynthesis, cysteine and methionine metabolism, and α-linolenic acid metabolism.

As phytohormone participated in many important steps in both Cd and melatonin response pathways, we then explored the expression pattern for genes in the pathway of plant hormone signal transduction, including auxin, cytokinine, gibberellin, abscisic acid, ethylene, and jasmonic acid, to identify the candidate gene related to Cd stress response and melatonin-mediating Cd detoxing. The genes in each signaling pathway display a variable expression pattern in different treatments and tissues (Figure 6).

In auxin signaling pathway, the gene expression level displayed divergent change in the roots and leaves, and most genes have been upregulated in melatonin treatment groups (Figure 6). On the contrary, we found one TIR1 copy, one ARF copy, and three SAUR copies that showed decreased RPKM values in melatonin samples.

In cytokinin signaling pathway, most genes showed stronger expression in the roots (Figure 6). In CRE1, the change in the expression pattern was different with melatonin treatment, while two copies were upregulated in melatonin-treatment samples and the other three copies were downregulated. A-ARR copies were upregulated by melatonin while the B-ARR showed the opposite expression pattern.

In gibberellin signaling pathway, three GID1 copies were downregulated by melatonin in the roots (Figure 6). In one GID1 copy, it has a stronger expression level in the leaves at three sampling timepoints. In the roots, its expression level reaches its peak on day 1, and its expression level was constrained by melatonin. In contrast, it showed the opposite pattern in the leaves in which the samples pre-treated by melatonin had higher RPKM values. GID2 demonstrates a remarkably higher expression level in the roots than leaves. We identified five DELLA-like genes that have significantly different expression between samples, and all of them were downregulated by melatonin on day 0, while four of them were only downregulated in the roots. The expression of TF-like gene was also upregulated by melatonin in the roots and downregulated in the leaves.

In abscisic acid signaling pathway, most copies of the early response gene PYR/PYL were downregulated by melatonin treatment on day 0 and day 1 (Figure 6). A SnRK2-like gene, 7_9141, was induced to highly express in root; we also found a homolog (1_21140) with a similar pattern in ABF.

In ethylene signaling pathway, we identified three SIMKK copies in lettuce differentially expressed between samples, and all of them showed a lower expression level in melatonin-treatment samples during day 0 and day 1 and a higher expression level on day 5 in the roots (Figure 6). We found two EIN2-like genes that displayed functional division since they have opposite expression pattern over time; one of them was upregulated and the other was downregulated by melatonin. Interestingly, the expression level of EBF1/2, EIN3, and ERF1/2 increased continuously over time in the leaves, while the melatonin treatment constrained the process, especially on day 5.

In jasmonic acid signaling pathway, we identified two copies of JAR1-like genes, 4_34281 and 7_2120, and both of them were downregulated in the roots with melatonin. In the leaves, the expression pattern was different (Figure 6). The expression of 4_34281 was downregulated by melatonin on day 0 and day 1 then showed an abnormally higher RPKM value on day 5. Furthermore, 7_2120 has a significantly higher RPKM value in the leaves after melatonin treatment. The COI1 was also downregulated by melatonin. JAZ genes show different expression patterns during treatment, which shows they have different functions. MYC2 genes also show different expression patterns between the roots and leaves. In the roots, there was a high expression level on day 0, decreasing on day 1 and increasing again on day 5, while the expression level of MYC2-like genes increased over time in the leaves.

## 4. Discussion

In the last decade, transcriptome sequencing has been applied as a novel technique to study how plants respond to Cd stress and how melatonin promotes the resistance processes. Studies focused on genotypes with different accumulation abilities to Cd also revealed the potential pathway related to the Cd resistance mediately. For example, in heavy-metal-hyperaccumulator *Sedum alfredii*, the expression level of genes involved in cell-wall modification, metal translocation, and remobilization were higher than the non-hyperaccumulating ecotype under Cd stress [48]. In rice, the WGCNA analysis demonstrated that the accumulation of Cd increased the expression level of chitinase as well as the genes related to heavy-metal transportation [49]. Although high-quality genomic research has been published for lettuce and facilitates the molecular breeding as well as its utility [50], the genetic data for Italian lettuce are still insufficient. Our understanding of the molecular background of Italian lettuce is limited, as is the new technological development base on molecular strategies. In this study, we provide the first reference transcriptome using single-molecule long-read sequencing complete-transcriptome analysis. Based on the high-quality reference, we annotated the new genes and potential novel alternative splicing of Italian lettuce. These novel genes are relevant to the unique feature for Italian lettuce compared to common lettuce. The prediction of novel genes may enrich the genome information of lettuce and offer a new direction to further research for its breeding. This study fills the genetic gaps in the molecular study of lettuce and facilitates further molecular breeding.

The expression patterns of vegetables are widely studied, while the Cd accumulation initials many molecular pathways, which are reflected in the high DEG numbers [51]. In this study, the numbers of DEGs in each comparing group show a large difference, reflecting the spatial and temporal expression of heterogeneity in the roots or leaves of Italian lettuce during Cd stress or Cd-and-melatonin treatment (Figure 3), and different stages and treatment group show totally different expression patterns and genes (Table S11). The different expression analysis in this study demonstrates that the roots and leaves have different response patterns under Cd stress with or without melatonin treatment. After Cd treatment, roots displayed a large amount of DEGs early on, and their number decreased on day 5. The aboveground part showed a slow response; the fiercest increase in the DEG number occurred in the late period, which formed a “malposed” peak compared to those in the roots. This result clearly reveals that the spatiotemporal accumulation for Cd is different between tissues in lettuce while the aboveground part showed a late response. In seedlings pre-applied melatonin, the situation was similar. In the roots, the number of DEGs had little difference between day 0 and 5, indicating that their response to Cd stress was continuous from day 0 to 5. Between day 0 and 1, the number of DEGs in the leaves was 171, increasing to 941 between day 1 and 5, and indicates a stronger response at late timepoints for the aboveground part. In common, Cd can be absorbed via both roots and leaves, but roots contribute to the main uptake of heavy metals [51]. Additionally, it was reported that Cd accumulation in the leaves was transported from the roots through phloem in lettuce [51]. Thus, the late response for the leaves can be attributed to the vacuum phase for the Cd translocation from the roots.

As expected, the Cd accumulation induced a high reactive-oxygen-species (ROS) level, and might damage the normally biological process of plant cells, including the toxic to metabolism, and key regulators of growth, development, and defense pathways [52]. The application of melatonin in plants protects plants from environmental stress, such as heavy metals, aging, and drought, as well as the process related to its antioxidant ability [18]. Cd stress leads to peroxidatic reaction; plants have evolved antioxidant defense mechanisms, such as peroxidase, to circumvent the damaging effects of ROS [38]. We also detected several DEGs enriched in terms “oxidation-reduction process” during Cd accumulation, indicating that these genes are involved in antioxidant defense mechanisms. In our study, the pathways involved in oxidoreductase activity were activated during Cd stress, and melatonin pretreatment actually induced several genes in this process, such as LOX1/5-like gene *5_148821*, and the POX-like genes *7_80360* and *Novelgene2763*. Nevertheless, the genes related to these pathways showed different expression levels among tissues and treatment, even the homolog genes. The result indicates that the genes related to antioxidants have a functional bias toward the leaves or roots, and that this bias is also toward the early or late stages after Cd treatment.

The effects of melatonin on plant-growth regulation can also enhance the stress resistance of plants to heavy-metal stress. In *Solanum lycopersicum*, the transcriptome analysis helps to identify a melatonin-induced pathway under sodic alkaline stress tolerance, in which the IAA3 in auxin signaling pathway and DREB1α contributed to the activation of several physiological processes of Na+ detoxification, dehydration resistance, and reactive-oxygen-species scavenging [53]. In the comparison study for the high- and low-Cd accumulating genotype of pakchoi (*Brassica chinensis*), it was reported that cell wall biosynthesis and glutathione metabolism were involved in Cd resistance in high-Cd accumulating genotype, while the genes related to DNA repair and ABA signal transduction pathways were upregulated in low-Cd accumulating genotype [12]. Melatonin pre-treatment downregulated the expression level of genes related to the jasmonic acid signaling pathway between day 0 and day 1. How the melatonin treatment alleviates the heavy-metal stress was also studied in many plant species. In the root of melon (*Cucumis melo*) under Cu stress, melatonin pretreatment changes the expression level of genes involved in redox and cell wall formation and also inhibits the biosynthesis of jasmonic acid biosynthesis [54].

In *Aeluropus littoralis* under salinity stress, the enriched GO terms of different expressed genes were the chitin response, response to abscisic acid, and regulation of jasmonic-acid-mediated signaling pathway [55]. The expression level for other plant hormone signal transduction pathways under sanity stress was also explored, and it was found that the AUX/IAA gene was downregulated and GH3 and SAUR were upregulated in auxin signaling pathway; in ABA, PYR/PYL gene was downregulated, while PP2C, SnRK2, and ABF were upregulated; in ethylene, EBF1/2 was downregulated, and JAZ in jasmonic acid signaling pathway was upregulated [55]. In wheat, the DEGs between low and high Cd accumulation were involved in ion binding, the antioxidant defense mechanism, sulfotransferase activity, and the cysteine biosynthetic process [56]. Thus, our study indicated that the hormone-signal-transduction pathways all participate in the alleviation process.

Plants respond to Cd stress via complicated signal transduction cascade, and these pathways always overlap and crosstalk. These processes have been proved to contain the phosphorylation of kinase such as MAPK, Ca-calmodulin system, ROS signaling, and plant hormones such as jasmonic acid and salicylic acid (SA) [57]. Through this, the expression pattern of corresponding genes changes over time and refers to the plant Cd-resistance progress. More than signal transduction cascade, the modulation of transcription factors is also activated to mediate the response to Cd stress, such as WRKY [58], basic leucine Zipper bZIP [59], ethylene-responsive factor ERF [60], and myeloblastosis protein MYB [61], which also activated during response to other abiotic stresses such as cold, dehydration, SA, and H_2_O_2_ [57]. Thus, the pathways related to ROS and plant hormones are also thought to respond to Cd and melatonin treatment. Further research focused on the connection between these pathways should be performed.

## 5. Conclusions

Our understanding of the molecular mechanism of Cd response in Italian lettuce and melatonin detoxification is still lacking, and the insufficiency of reference transcriptome and whole-genome assembly represents the main barrier. Based on single-molecule sequencing and illumine technology, we obtained the full-length transcriptome as well as the expression profile of Italian lettuce under Cd stress and melatonin treatment over time, which could be used as the reference omics data for the study of lettuce. We found that the molecular pathway of Cd response first occurs in the roots, and the leaves demonstrate hysteresis. The response in the roots is also much stronger compared to the aboveground part. We then found that genes related to antioxidant and plant hormone signal transduction display a functional division, in which those show totally different expression levels between the roots and leaves across three studied time points. We then identified genes related to the melatonin detoxify mechanism in Italian lettuce, which will act as the future candidates for molecular breeding.

## Figures and Tables

**Table 1:** Differential gene statistics in six comparison groups.

Comparing Group	Control	Treatment	Up	Down	Total DEG Number
G1	LCd0	LCd1	360	418	778
G1	LCd1	LCd5	706	777	1483
G2	LCdM0	LCdM1	92	81	173
G2	LCdM1	LCdM5	405	536	941
G3	LCd0	LCdM0	7	24	31
G3	LCd1	LCdM1	0	0	0
G3	LCd5	LCdM5	7	10	17
G4	LCd0	LCd1	752	994	1746
G4	LCd1	LCd5	282	192	474
G5	LCdM0	LCdM1	177	327	504
G5	LCdM1	LCdM5	245	84	329
G6	RCd0	RCdM0	704	436	1140
G6	RCd1	LCdM1	2178	1349	3527
G6	LCd5	LCdM5	0	0	0

G1–G6 represent comparison groups 1–6, respectively. Leaf samples on days 0, 1, and 5 under cadmium stress are represented by LCd0, LCd1, and LCd5. Leaf samples on days 0, 1, and 5 under cadmium stress that were pre-treated by melatonin are represented by LCdM0, LCdM1, and LCdM5. Root samples on days 0, 1, and 5 under cadmium stress are represented by RCd0, RCd1, and RCd5. Root samples on days 0, 1, and 5 under cadmium stress that were pre-treated by melatonin are represented by RCdM0, RCdM1, and RCdM5.

## Data Availability

Not applicable.

## Supplementary Materials

The following supporting information can be downloaded at: https://www.mdpi.com/article/10.3390/genes13060955/s1, Figure S1: Pearson’s correlation coefficient for correlation analysis for all samples; Table S1: Sample information; Table S2: Sequencing information; Table S3: Leaf and root new gene and annotation; Table S4: Leaf and root new isoform; Table S5: S Leaf and root AS total; Table S6: Fusion genes; Table S7: Leaf and root.cpc; Table S8: Leaf and root.cnci; Table S9: Leaf and root.pfam; Table S10: Leaf and root.plek; Table S11: Enrichment-GO-KEGG; Table S12: Enrichment for Group1; Table S13: Enrichment for Group4; Table S14: Enrichment for Group2; Table S15: Enrichment for Group5; Table S16: Enrichment for Group3; Table S17: Enrichment for Group6.
