# Supplementary material for: Comparative Analysis of Italian Lettuce (Lactuca sativa L. var. ramose) Transcriptome Profiles Reveals the Molecular Mechanism on Exogenous Melatonin Preventing Cadmium Toxicity
Source: Genes (Basel). 2022 May 26;13(6):955. doi: 10.3390/genes13060955 (PMC9223142; doi:10.3390/genes13060955)
Supplement: Supplementary file 1 [file genes-13-00955-s001.zip › Supplementary Files/Figure S1_Pearson's correlation coefficient for correlation analysis for all samples.pdf]

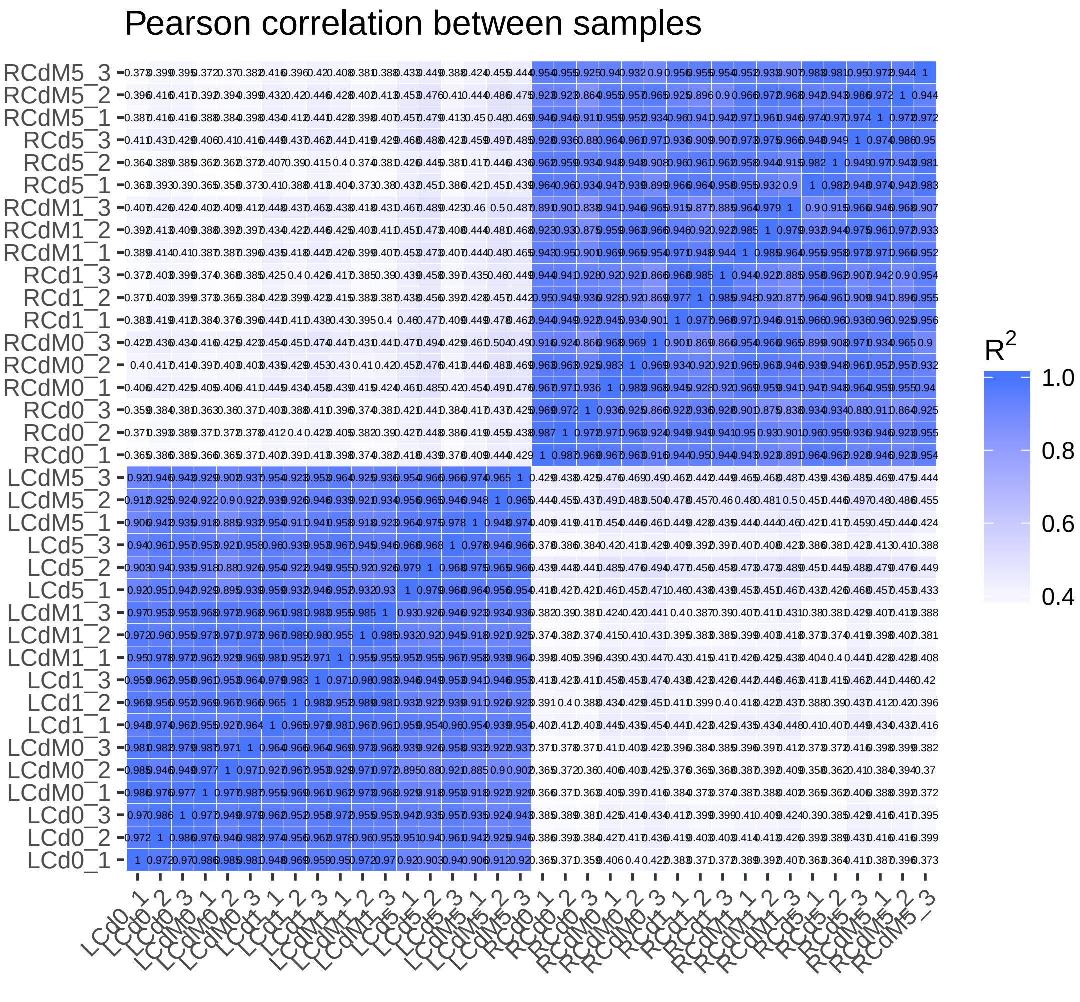
Pearson correlation between samples
RCdM5_3
RCdM5_2
RCdM5_1
RCd5_3
RCd5_2
RCd5_1
RCdM1_3
RCdM1_2
RCdM1_1
RCd1_3
RCd1_2
RCd1_1
RCdM0_3
RCdM0_2
RCdM0_1
RCd0_3
RCd0_2
RCd0_1
LCdM5_3
LCdM5_2
LCdM5_1
LCd5_3
LCd5_2
LCd5_1
LCdM1_3
LCdM1_2
LCdM1_1
LCd1_3
LCd1_2
LCd1_1
LCdM0_3
LCdM0_2
LCdM0_1
LCd0_3
LCd0_2
LCd0_1
R2
1.0
0.8
0.6
0.4
